# Physical therapies for Achilles tendinopathy: systematic review and meta-analysis

**DOI:** 10.1186/1757-1146-5-15

**Published:** 2012-07-02

**Authors:** Samuel P Sussmilch-Leitch, Natalie J Collins, Andrea E Bialocerkowski, Stuart J Warden, Kay M Crossley

**Affiliations:** 1 Department of Physiotherapy, The University of Melbourne, Melbourne, VIC, Australia; 2 Department of Mechanical Engineering, Melbourne School of Engineering, University of Melbourne, Melbourne, VIC, Australia; 3 Department of Physiotherapy, University of Western Sydney, School of Biomedical and Health Sciences, Campbelltown, NSW, Australia; 4 Department of Physical Therapy, School of Health and Rehabilitation Sciences, Indiana University, Indianapolis, USA; 5 School of Health and Rehabilitation Sciences, University of Queensland, Brisbane, QLD, Australia

**Keywords:** Achilles tendon, Tendinopathy, Physical therapy modalities

## Abstract

**Background:**

Achilles tendinopathy (AT) is a common condition, causing considerable morbidity in athletes and non-athletes alike. Conservative or physical therapies are accepted as first-line management of AT; however, despite a growing volume of research, there remains a lack of high quality studies evaluating their efficacy. Previous systematic reviews provide preliminary evidence for non-surgical interventions for AT, but lack key quality components as outlined in the Preferred Reporting Items for Systematic Reviews and Meta-analyses (PRISMA) Statement. The aim of this study was to conduct a systematic review and meta-analysis (where possible) of the evidence for physical therapies for AT management.

**Methods:**

A comprehensive strategy was used to search 11 electronic databases from inception to September 2011. Search terms included *Achilles, tendinopathy, pain, physical therapies, electrotherapy* and *exercise* (English language full-text publications, human studies). Reference lists of eligible papers were hand-searched. Randomised controlled trials (RCTs) were included if they evaluated at least one non-pharmacological, non-surgical intervention for AT using at least one outcome of pain and/or function. Two independent reviewers screened 2852 search results, identifying 23 suitable studies, and assessed methodological quality and risk of bias using a modified PEDro scale. Effect size calculation and meta-analyses were based on fixed and random effects models respectively.

**Results:**

Methodological quality ranged from 2 to 12 (/14). Four studies were excluded due to high risk of bias, leaving 19 studies, the majority of which evaluated midportion AT. Effect sizes from individual RCTs support the use of eccentric exercise. Meta-analyses identified significant effects favouring the addition of laser therapy to eccentric exercise at 12 weeks (pain VAS: standardised mean difference −0.59, 95% confidence interval −1.11 to −0.07), as well as no differences in effect between eccentric exercise and shock wave therapy at 16 weeks (VISA-A:–0.55,–2.21 to 1.11). Pooled data did not support the addition of night splints to eccentric exercise at 12 weeks (VISA-A:–0.35,–1.44 to 0.74). Limited evidence from an individual RCT suggests microcurrent therapy to be an effective intervention.

**Conclusions:**

Practitioners can consider eccentric exercise as an initial intervention for AT, with the addition of laser therapy as appropriate. Shock wave therapy may represent an effective alternative. High-quality RCTs following CONSORT guidelines are required to further evaluate the efficacy of physical therapies and determine optimal clinical pathways for AT.

## Background

Achilles tendinopathy (AT) is the generic descriptor used to describe the clinical presentation of activity-related Achilles tendon pain, focal tendon tenderness and intratendinous imaging changes. It is a common condition causing considerable morbidity in athletes and non-athletes alike [1,2]. Symptoms can occur at the midportion or insertion of the tendon, with the underlying pathology reflecting a failed healing response [3,4], where both inflammatory and degenerative pathologies exist. Histology studies indicate that the pathology is predominantly of tendon degeneration (‘tendinosis’) as opposed to the historically hypothesised inflammation (‘tendinitis’) [5-7] and can develop long before the onset of symptoms. This may result in advanced underlying pathology prior to clinical presentation, which has repercussions for management, as well as outcome expectations of both the clinician and patient. It also may partly explain why some individuals develop recalcitrant AT [8] and may progress to full tendon rupture [9].

Conservative or physical therapies are generally accepted as the first line approach for managing AT [10-12], and can be used in isolation or in conjunction with pharmacological and injectable agents. Surgical approaches are usually reserved for the most recalcitrant cases. Physical therapies for AT include exercise, electrotherapeutic modalities, soft tissue therapies, braces and splints. These are often used in a multimodal approach for the purpose of alleviating symptoms and promoting functional recovery.

Although the evidence base for physical therapies for AT continues to evolve, there remains a lack of evidence for their efficacy from high-quality studies. McLauchlan and Handoll [13] performed the first systematic review of randomised controlled trials (RCTs) for AT interventions, identifying nine eligible studies. The authors concluded there to be insufficient evidence to recommend any intervention for the management of AT. More recent systematic reviews examined non-surgical treatment of midportion [14] and insertional [11] AT. Magnussen et al. [14] reported that eccentric exercises had the most evidence for their efficacy in treating midportion AT, but the authors were not able to conduct a meta-analysis due to heterogeneity between treatment groups. Kearney and Costa [11] restricted the scope of their systematic review to studies of insertional AT, which limited the number of RCTs retrieved to one. To compensate, they included all other study designs other than single case studies and, as a result, their conclusions were based on studies lower on the hierarchy of scientific evidence. Nevertheless, the authors reported a lack of evidence regarding interventions for insertional AT.

Previous systematic reviews for the conservative management of AT provide useful summaries of the available evidence; however, they lack key quality components of systematic reviews as outlined in the Preferred Reporting of Systematic Reviews and Meta-Analyses (PRISMA) statement [15]. Notably, none of the reviews conducted methodological quality assessment of the included studies, calculated effect sizes or performed meta-analyses. Considering this, and recent increases in research output in the field, it is timely to provide an updated synthesis of the evidence for non-surgical, non-pharmacological management options for AT. The aim of this study was to conduct a systematic review and meta-analysis (where possible) of the evidence for physical therapies for the management of AT.

## Methods

The study design was developed in consultation with PRISMA guidelines [15].

### Eligibility criteria

Studies eligible for inclusion were RCTs evaluating the effect of at least one non-surgical, non-pharmacological intervention on pain and/or altered function associated with AT. Achilles tendinopathy was defined as participants experiencing one or more common signs or symptoms (tenderness on palpation, pain at rest or during activity, stiffness during activity, and impaired function), either in the midportion or insertional region of the Achilles tendon. The diagnosis of AT had to be made by a healthcare or medical practitioner. No restrictions were placed on the duration of participant symptoms, or length of treatment or follow up period. Studies were excluded if they included results that had been reported in previous publications, or if they included participants with symptoms related to Achilles rupture, rheumatological disease or the use of fluoroquinolone antibiotics. The search was limited to studies available in full-text, written in English and evaluating human participants.

### Identification of studies

A comprehensive search strategy was developed using the National Health and Medical Research Council guidelines [16]. Medline, EMBASE, Web of Science, Cumulative Index to Nursing and Allied Health Literature (CINAHL), Health and Medical Complete, Proquest, Australian Medical Index (AMI), Australian Sport Database (AUSPORT), AUSPORT Medical, Physiotherapy Evidence Database (PEDro), and Clinical Evidence databases were searched from their earliest record to September 13^th^ 2011. The search strategy for Medline ( Additional file 1) was adapted for use in the other databases. Secondary searching was conducted by reviewing reference lists of eligible papers.

Titles, abstracts and full text articles, where necessary, were screened for eligibility by two independent reviewers (KC and SW). Discrepancies were discussed in a consensus meeting and the opinion of a third independent reviewer (AB) was sought if agreement could not be achieved.

### Methodological quality assessment

A modified version of the Physiotherapy Evidence Database (PEDro) scale [17] was used to assess the methodological quality of included studies ( Additional file 2). Three additional criteria were added to the existing 11 PEDro criteria to evaluate sample size, validity and reliability of outcome measures, and reporting of adverse or side effects [18]. One point was awarded for each criterion that was clearly satisfied according to prespecified guidelines, and the 14 items summed to give a total methodological quality score out of 14. The modified PEDro scale has been used in previous systematic reviews [18-20] and has good inter-rater reliability (κ 0.73 to 0.82) [18]. Two reviewers (SSL and AB) completed formal training for using the PEDro scale [17] and independently rated each eligible study. A consensus meeting was held to resolve any discrepancies between the reviewers. When the two reviewers could not reach agreement, a third independent reviewer was consulted (SW).

The risk of bias was established for each study, using specific criteria from the modified PEDro scale. These were chosen after consulting the PRISMA Statement [15] as well as recommendations made by the Cochrane Collaboration [21]. Six criteria were used in the assessment: i) adequacy of randomisation (criterion two); ii) allocation concealment (criterion three); iii) between-group baseline comparability (criterion four); iv) blinding of outcome assessors (criterion seven); v) adequate follow-up (more than 85%) (criterion eight), and; vi) intention to treat analysis (criterion nine). A score of five or six was considered to have a low risk of bias, three to four a moderate risk, and two or less a high risk. Studies that had a high risk of bias were excluded from further analyses.

### Data extraction and analysis

The kappa (κ) statistic was used to calculate the inter-rater reliability of the modified PEDro scores. The magnitude of agreement was defined as per Hopkins [22], where 0.9 to 1.0 represented almost perfect to perfect agreement, 0.7 to 0.9 very high agreement, 0.5 to 0.7 high agreement, 0.3 to 0.5 moderate agreement, 0.1 to 0.3 small agreement, and 0.0 to 0.1 very small agreement.

Data extraction was performed by one author (SSL) and included participant characteristics, diagnostic criteria, AT characteristics, interventions, outcome measures and outcome data. For studies that utilised more than one outcome measure for pain and/or function, outcome data for a disease-specific outcome of pain and/or function was extracted. If this was not possible, a commonly-used outcome measure was chosen (e.g. pain visual analogue scale). If insufficient data were presented for calculation of effect sizes, an attempt was made to electronically contact the corresponding author for further information. Calculations of mean differences (SMD) and 95% confidence intervals (CI) were generated by Review Manager software [23] using an inverse variance method and fixed effects model for individual studies. Where studies had sufficient homogeneity in participant characteristics, interventions, outcome measures and follow up times, a meta-analysis of outcome data was performed. Meta-analyses were conducted using a random effects model, as some pooled studies had a heterogeneity greater than 50%, which was determined using an I^2^ statistical assessment of inconsistency [24]. Interpretation of SMDs was conducted as per Hopkins [22], where an effect size of 4.0 was considered to represent an extremely large clinical effect, 2.0 to 4.0 a very large effect, 1.2 to 2.0 a large effect, 0.6 to 1.2 a moderate effect, 0.2 to 0.6 a small effect, and 0.0 to 0.2 a trivial effect. Negative values favoured the intervention of interest and a null effect was considered for 95% CIs that contained zero.

## Results

**Figure 1:**
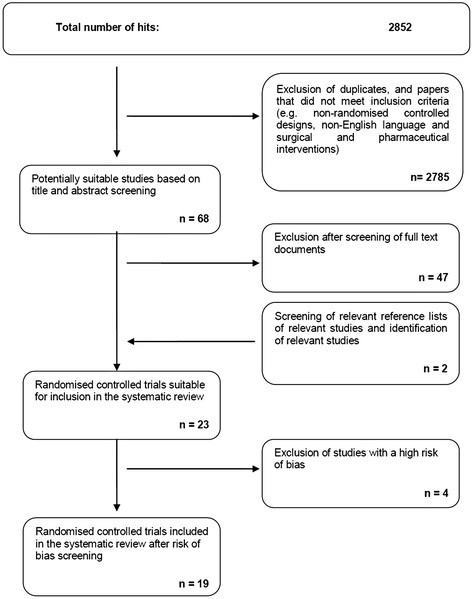
Flow chart of the process and rationale used in selecting studies for inclusion.

The search strategy identified 2852 studies, of which 68 required further full-text screening (Figure 1). Twenty-three primary studies met the inclusion criteria. The studies were conducted across nine countries: Germany [25-31], United Kingdom [32-36], Sweden [37-40], Denmark [41,42], New Zealand [43], Northern Ireland [44], Norway [45], Canada [46] and The Netherlands [47].

### Methodological quality

The two reviewers had initial agreement on 297 out of 322 criteria (κ = 0.941, 95% CI 0.904 to 0.978) (Table 1), and reached consensus on all criteria. The inter-rater reliability for individual criteria ranged from high to perfect (κ = 0.621 to 1.000). Quality assessment scores ranged between two and 12 out of a maximum of 14 (mean ± SD 7.8 ± 2.9). Reporting of random group allocation and the results of between-group statistical comparisons were scored by all studies. Criteria that were met by the least number of studies were blinding of therapists (one study), and reliability and validity of outcome measures (two studies). Four studies were considered to have a high risk of bias [28,36,37,41], and were subsequently excluded from further analyses, leaving 19 studies remaining.

**Table 1:** Quality ratings and inter-rater reliability using the Modified PEDro Scale of reviewed studies (N = 23)

	**Criteria**														
	**1**	**2**	**3**	**4**	**5**	**6**	**7**	**8**	**9**	**10**	**11**	**12**	**13**	**14**	** *Total* **
Costa 2005	✓	✓	✓	✓	✓		✓	✓	✓	✓	✓	✓		✓	**12**
Tumilty 2008	✓	✓	✓		✓	✓	✓	✓	✓	✓	✓	✓	✓		**12**
Rasmussen 2008	✓	✓	✓	✓	✓		✓	✓	✓	✓	✓	✓			**11**
Rompe 2007	✓	✓	✓	✓			✓	✓	✓	✓	✓	✓		✓	**11**
Rompe 2008	✓	✓	✓	✓			✓	✓	✓	✓	✓	✓		✓	**11**
Rompe 2009	✓	✓	✓	✓			✓	✓	✓	✓	✓	✓		✓	**11**
Silbernagel 2007	✓	✓	✓	✓			✓	✓	✓	✓	✓	✓			**10**
Stergioulas 2008	✓	✓		✓	✓		✓	✓	✓	✓	✓			✓	**10**
de Jonge 2010	✓	✓	✓	✓			✓	✓	✓	✓		✓			**9**
Chapman-Jones 2002	✓	✓	✓	✓				✓		✓	✓	✓			**8**
Herrington 2007	✓	✓	✓	✓			✓	✓		✓				✓	**8**
Mafi 2001	✓	✓	✓						✓	✓	✓	✓		✓	**8**
Chester 2008	✓	✓	✓				✓			✓	✓			✓	**7**
** *Roos 2004* **	✓	✓							** *✓* **	** *✓* **	** *✓* **	** *✓* **		** *✓* **	** *7* **
Knobloch 2007		✓		✓			✓	✓	✓	✓			✓		**7**
Knobloch 2008	✓	✓					✓	✓		✓	✓				**6**
McAleenan 2010	✓	✓	✓							✓			✓	✓	**6**
Petersen 2007	✓	✓		✓				✓		✓	✓				**6**
Silbernagel 2001	✓	✓					✓	✓		✓	✓				**6**
** *Mayer 2007* **	✓	✓						**✓**			**✓**				** *4* **
Niesen-Vertommen 1992		✓		✓				✓		✓					**4**
** *Norregaard 2007* **	✓	✓								**✓**	**✓**				** *4* **
** *Lowdon 1984* **	✓	✓													** *2* **
**Inter-rater reliability (κ)**	**0.62**	**1.00**	**1.00**	**0.73**	**1.00**	**1.00**	**0.90**	**0.88**	**1.00**	**0.62**	**1.00**	**1.00**	**1.00**	**1.00**	**0.94**

Listing in descending order of quality rating.

Ticks indicate where a point was awarded for the criterion. Studies highlighted with bold and italic font were rated as having a high risk of bias.

### Participant characteristics

Characteristics of study participants are presented in Table 2. Most studies utilised chronic cohorts (symptoms greater than three months), with the minimum duration of symptoms ranging from six weeks to 12 months. Nine studies (47%) [29-31,33-35,38,40,42] included participants with symptom durations of three or more months. Clinical examination was the only diagnostic tool in 13 studies (68%) [25-27,29,31-33,35,39,42,44,45,47], while ultrasonography was considered in six studies (33%) [25-27,31,40,45]. Two-thirds of studies (67%) evaluated participants with only midportion symptoms, two studies (11%) [29,32] included participants with mixed diagnoses of insertional or midportion AT, while the location was not reported in four studies [34,42,44,46]. Seven studies (37%) [26,29,33,34,44-46] included participants with a mean age of 40 years or less, and six studies (32%) [25-27,32,35,42] utilised groups with at least 50 percent females.

**Table 2:** Participant characteristics

**Study**	**Type**	**Diagnosis**	**Sample size**	**Female (%)**	**Age (years) Mean (SD)**	**Pain duration (months), mean, range**
**ECCENTRIC EXERCISE**						
Silbernagel 2001	M	C	A: 22	A: 5 (23)	A: 47 (15)	A: 9, 7, 4–96
			B: 18	B: 4 (22)	B: 41 (10)	B: 18,13, 6-192
Chester 2008	M	C	A: 8	A: 4 (50)	A: 59 (10)	A: 23,13, NR
			B: 8	B: 1 (13)	B: 48 (12)	B: 14,10, NR
Mafi 2001	M	C & US	A: 22	A: 10 (45)	A:48 (10)	A: 18, NR, 3–120
			B: 22	B: 10 (45)	B: 48 (8)	B: 23, NR, 5-120
Rompe 2007	M	C & US	A: 25	A: 16 (64)	A: 48 (10)	A: 11, 8, NR
			B: 25	B: 16 (64)	B: 46 (11)	B: 9, 11, NR
Herrington 2007	M	C	A: 13	NR	A: 37 (9)	A: 21,18, NR
			B: 12		B: 37 (7)	B: 28,13, NR
Knobloch 2007	All	C	A: 15	A: 7 (47)	A: 33 (12)	A: NR
			B: 5	B: 2 (40)	B: 32 (10)	B: NR
Niesen-Vertommen 1992	NR	NR	A: 8	A: 4 (50)	A: 35 (NR)	A: 4, NR, NR
			B: 9	B: 3 (33)	B: 34 (NR)	B: 4, NR, NR
Petersen 2007	M	C & US	A: 37	A: 14 (38)	A: 42 (11)	A: 7, 3, NR
			B: 35	B: 15 (43)	B: 42 (11)	B: 7, 3, NR
**SHOCK WAVE THERAPY**						
Rasmussen 2008	NR	C	A: 24	A: 12 (50)	A: 49 (9)	A: NR
			B: 24	B: 16 (67)	B: 46 (13)	B: NR
Costa 2005	All	C	A: 22	A: 13 (59)	A: 58 (11)	A: 18,10, NR
			B: 27	B: 15 (56)	B: 47 (13)	B: 21, 21, NR
Rompe 2007	M	C & US	A: 25	A: 14 (56)	A: 51 (10)	A: 13, 7, NR
			B: 25	B: 16 (64)	B: 46 (11)	B: 9, 11, NR
			C: 25	C: 16 (64)	C: 48 (10)	C: 11, 8, NR
Rompe 2008	I	C & US	A: 25	A: 16 (64)	A: 40 (11)	A: 26,11, NR
			B: 25	B: 14 (56)	B: 39 (11)	B: 25, 8, NR
Rompe 2009	M	C & US	A: 34	A: 18 (53)	A: 53 (10)	A: 16, 5, NR
			B: 34	B: 20 (59)	B: 46 (10)	B: 13, 7, NR
**NIGHT SPLINT**						
de Jonge 2010	M	C	A: 36	A: 14 (39)	A: 45 (9)	A: 28, 46, NR
			B: 34	B: 12 (35)	B: 44 (7)	B: 34, 56, NR
McAleean 2010	NR	C	A: 5	A: 2 (40)	A: 42 (6)	A 11, 14, NR
			B: 6	B: 3 (50)	B: 40 (9)	B: 19, 12, NR
**HEEL BRACE**						
Knobloch 2008	M	C	A: 43	A: 14 (33)	A: 47 (11)	A: NR
			B: 54	B: 20 (37)	B: 48 (11)	B: NR
Petersen 2007	M	C & US	A: 28	A: 11 (39)	A: 43 (12)	A: 7, 2, NR
			B: 37	B: 14 (38)	B: 42 (11)	B: 7, 3, NR
**LASER THERAPY**						
Stergioulas 2008	M	C	A: 20	A: 8 (40)	A: 30 (5)	A: 10, 3, NR
			B: 20	B: 7 (35)	B: 29 (5)	B: 9, 3, NR
Tumilty 2008	M	NR	A: 10	A: 3 (33)	A: 41 (7.6)	A: 4, NR, NR
			B: 10	B: 6 (60)	B: 43 (8.5)	B: 4, NR, NR
**MICROCURRENT THERAPY**						
Chapman-Jones 2002	NR	C	A: 24	A: 6 (25)	A: 39 (10.4)	A: NR
			B: 24	B: 7 (29)	B: 36 (7.8)	B: NR
**CONTINUED TENDON LOADING**						
Silbernagel 2007	M	C	A: 26	A: 7 (37)	A: 44 (8.8)	A: 48, 85, 3–360
			B: 25	B: 11 (58)	B: 48 (6.8)	B: 24, 41, 3-168

All = includes both I & M; C = clinical; I = insertional; M = midportion; NA = not applicable; NR = not reported; US = ultrasound.

### Outcome measures

Six different measures of pain and/or function were reported across the 19 studies, with the evaluation of pain-only being most common (79% of studies). Combined measures of pain and function (47% of studies), and function-only measures (21% of studies) were also used. Visual analogue scales (VAS) (79% of studies) [25-27,29-32,35,39,40,42,43,45,46] and the Victorian Institute of Sport Assessment–Achilles (VISA-A) questionnaire (37% of studies) [25,26,33,39,43,44,47] were the most frequently used tools. The American Orthopedic Foot and Ankle Society hindfoot scale (AOFAS) (11%) [31,42], Functional Index of the Leg and Lower Limb (FILLA) (11%) [32,35], Pain Scoring System (5%) [34], General Assessment of function (5%) [34] and Heel-raise test (5%) [44] were also used. Reliability was reported for three outcome measures (VAS [48], VISA-A [49] and FILLA [50]) and only one measure had reported validity (VISA-A [49]). Additional data was requested for six of the 19 studies [33,34,38,42,46,47], with three authors replying to correspondence [33,38,47], and one providing sufficient data for further evaluation [47].

### Evidence for physical therapies

**Figure 2:**
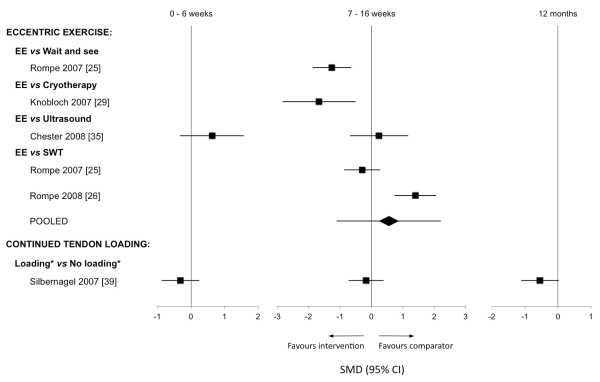
**Standardised mean differences for outcomes of pain ± function following intervention with exercise modalities.** EE = eccentric exercise; SWT = shock wave therapy. * denotes use of conservative therapy in addition to presented modality.

*Exercise modalities:* Eccentric exercise was the most frequently investigated intervention (17 out of the 19 studies). Nine studies investigated eccentric exercise programs as a primary intervention of interest [25,26,29,31,33,35,38,40,46]. A further eight studies used eccentric exercise as a control or adjunct intervention [27,30,34,39,42,43,45,47], and these will be considered under their respective primary interventions. The methodological quality of the nine studies ranged from four to 11 out of 14 (mean ± SD 7.6 ± 5.3). Four of these studies [25,26,29,35] provided sufficient data for effect size calculation (Figure 2). Due to differences in comparator interventions, outcome measures and follow up times, pooling of data from these studies was not conducted.

Only one study compared eccentric exercise to a wait-and-see control [25], with findings of large significant effects favouring a 12-week eccentric exercise program (SMD −1.26, 95% CI −0.65 to −1.87). Similar effects were also seen when 12 weeks of eccentric exercise was compared to cryotherapy (−1.67,–0.50 to −2.83) [29]. In contrast, Petersen et al. [31] reported no significant differences in outcome over one year between those treated with 12 weeks of eccentric exercise and a heel brace (*p* > 0.05).

Three studies compared eccentric exercise to electrotherapeutic modalities. Effect sizes for Chester et al. [35] showed that eccentric exercise was not significantly different to therapeutic ultrasound at six weeks (0.63,–0.33 to 1.58) and 12 weeks (0.24,–0.69 to 1.17). Two studies compared 12 weeks of eccentric exercise to three weeks of shock wave therapy (SWT) [25,26], with pooled data showing no significant differences at 16 weeks (VISA-A:–0.55,–2.21 to 1.11).

A 12-week eccentric exercise program was compared directly to concentric exercise by two studies [40,46]. Although effect sizes could not be calculated for either study, Niesen-Vertommen et al. [46] reported significantly greater pain reduction in the eccentric exercise group at four, eight and 12 weeks (*p* < 0.05). Mafi et al. [40] did not present between-group comparisons for pain outcomes at 12 weeks. Silbernagel et al. [38] compared two rehabilitation programs, both using eccentric and concentric calf strengthening. Those randomised to the experimental group received a program of higher Achilles tendon loading that induced higher pain levels than the control program, although they received greater therapist monitoring than the control group. However, no conclusions could be drawn regarding comparative efficacy, due to the absence of between-group comparisons of pain, and effect estimates could not be calculated due to insufficient data.

Herrington and McCulloch [33] assessed the benefit of adding eccentric exercise to a multimodal program of deep friction massage, ultrasound and calf stretching. Pain and function outcomes assessed using the VISA-A questionnaire suggest that the eccentric exercise group experienced greater improvements after 12 weeks than the control group (*p* = 0.01); however, effect estimates could not be calculated due to insufficient data.

Silbernagel et al. [39] evaluated the effect of continued tendon loading while undergoing a rehabilitation program of eccentric exercises for AT. One group continued to participate in tendon loading activities (e.g. running or jumping activities) while the other limited this type of activity, and groups were followed over one year. Evaluation of pain and function outcomes found no significant effects for either program at six weeks (−0.32, -0.88 to 0.25), 12 weeks (−0.17,–0.73 to 0.39), 26 weeks (−0.12,–0.68 to 0.44) or one year (−0.55,–1.11 to 0.02) (Figure 2).

**Figure 3:**
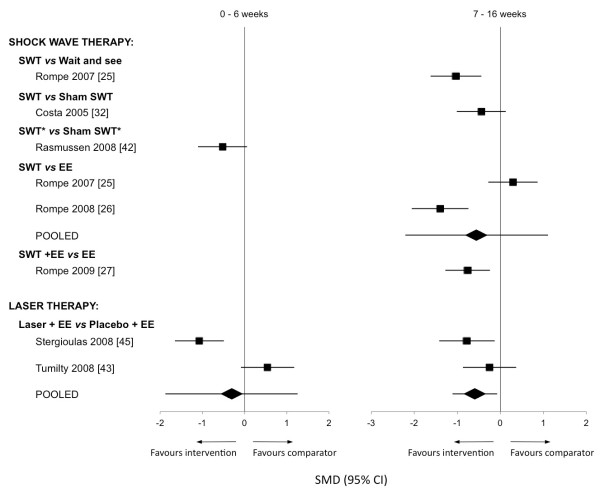
**Standardised mean differences for outcomes of pain ± function following electrophysical therapies.** SWT = shock wave therapy; EE = eccentric exercise. * denotes use of rehabilitation program in addition to presented modality.

*Electrophysical therapies:* Five studies [25-27,32,42] evaluated the efficacy of SWT on AT (Table 3), with a mean methodological quality of 11.2 ± 0.4 (range 11 to 12). Sufficient data for effect size calculations was available for all studies (Figure 3).

**Table 3:** Physical therapies for Achilles tendinopathy

**Study**	**Intervention(s)**	**Sample size**	**Intervention duration (wk)**	**Comparison and outcome measure**	**SMD (95% CI)**	**Study conclusions (where SMD unable to be calculated)**
**ECCENTRIC EXERCISE**						
Mafi 2001	A: Eccentric exercise	A: 22	12	A vs B	12wk: ID	Between groups comparisons of pain not presented; Significant within-group improvement in pain VAS for both eccentric and concentric exercise in those who were satisfied with treatment (*p* < 0.05)
	B: Concentric exercise	B: 22		VASa		
Niesen-Vertommen 1992	A: Eccentric exercise	A: 8	12	A vs B	4wk: ID	Eccentric exercise had a greater reduction of pain (p < 0.01)
	B: Concentric exercise	B: 9		VASo	8wk: ID	
					12wk: ID	
Rompe 2007	A: Eccentric exercise	A: 25	12	A vs B	16wk:–1.26 (–1.87:–0.65)	
	B: Wait and see approach	B: 25		VISA-A		
Knobloch 2007	A: Eccentric exercise	A: 15	12	A vs B	12wk: -1.67 (−2.83: -0.50)	
	B: Cryotherapy	B: 5		VASo		
Petersen 2007	A: Eccentric exercise	A: 37	12	A vs B	6wk: ID	No difference between groups (p < 0.05)
	B: Heel brace	B: 35		VASa	12wk: ID	
					54wk: ID	
Rompe 2008	A: Shock wave therapy	A: 25	A: 3	B vs A	16wk: -1.40 (−0.74: -2.06)	
	B: Eccentric exercise	B: 25	B: 12	VISA-A		
Chester 2008	A: Eccentric exercise	A: 8	A: 12	A vs B	6wk: 0.63 (−0.33: 1.58)	
	B: Ultrasound	B: 8	B: ≤6	VASs	12wk: 0.24 (−0.69: 1.17)	
Silbernagel 2001	A: Rehabilitation programme including single leg eccentric loading	A: 22	12	A vs B	6wk: ID	Eccentric loading had better strength and pain outcomes (p < 0.05)
		B: 18		VASj	12wk: ID	
	B: Rehabilitation programme				26wk: ID	
					52wk: ID	
Herrington 2007	A: Eccentric exercise + deep friction massage + ultrasound + calf stretches	A: 13	12	A vs B	4wk: ID	Eccentric exercise produced superior pain and function outcomes (p = 0.01)
	B: Deep friction massage + ultrasound + calf stretches	B: 12		VISA-A	8wk: ID	
					12wk: ID	
**SHOCK WAVE THERAPY**						
Costa 2005	A: Shock wave therapy	A: 22	12	A vs B	12wk: -0.44 (−1.01: 0.13)	
	B: Sham shock wave therapy	B: 27		VASw	52 wk: ID	
Rompe 2007	A: Shock wave therapy	A: 25	A: 3	A vs B	16wk: -1.03 (−1.62:-0.44)	
	B: Wait and see approach	B: 25	B: 12	VISA-A		
	C: Eccentric exercise	C: 25	C: 12	A vs C	16 wk: 0.29 (−0.27: 0.85)	
				VISA-A		
Rompe 2008	A: Shock wave therapy	A: 25	A: 3	A vs B	16wk: -1.40 (−2.03: -0.78)	
	B: Eccentric exercise	B: 25	B: 12	VISA-A		
Rompe 2009	A: Shock wave therapy + eccentric exercise	A: 34	A: 12	A vs B	16wk: -0.76 (−1.28: -0.24)	
	B: Eccentric exercise	B: 34	B: 12	VISA-A		
Rasmussen 2008	A: Shock wave therapy + conservative therapy	A: 24	4	A vs B	4wk: -0.52 (−1.10: 0.06)	
	B: Sham shock wave therapy + conservative therapy	B: 24		AOFAS	8wk: ID	
					12wk: ID	
**LASER THERAPY**						
Stergioulas 2008	A: Laser therapy + eccentric exercise	A: 20	8	A vs B	4wk: -1.07 (−1.65: -0.49)	
	B: Placebo laser therapy + eccentric exercise	B: 20		VASa	8wk: -1.14 (−1.82: -0.47)	
					12wk: -0.78 (−1.42: -0.13)	
Tumilty 2008	A: Laser therapy + eccentric exercise	A: 10	12	A vs B	4wk: 0.53 (−0.36: 1.43)	
	B: Placebo laser therapy + eccentric exercise	B: 10		VASm	12wk: -0.25 (−1.13: 0.64)	
**MICROCURRENT THERAPY**						
Chapman-Jones 2002	A: Microcurrent therapy + eccentric exercise	A: 24	12	A vs B	12wk: ID	Microcurrent therapy produced superior pain, stiffness and function outcomes (p < 0.001)
	B: Eccentric exercise^11^	B: 24		VASa	26wk: ID	
					52wk: ID	
**CONTINUED TENDON LOADING**						
Silbernagel 2007	A: Rehabilitation programme + continued tendon loading activity	A: 26	12- 26	A vs B	6wk: -0.32 (−0.88: 0.25)	
	B: Rehabilitation programme + no tendon loading activity (running or jumping)	B: 25		VISA-A-S	12wk: -0.17 (−0.73: 0.39)	
					26wk: -0.12 (−0.68: 0.44)	
					52wk: -0.55 (−1.11: 0.02)	
**NIGHT SPLINT**						
de Jonge 2010	A: Night splint + eccentric exercise	A: 36	12	A vs B	4wk: -0.12 (−0.61: 0.37)	
	B: Eccentric exercise	B: 34		VISA-A	12wk: 0.07 (−0.43: 0.56)	
					52wk: -0.10 (−0.60: 0.40)	
McAleenan 2010	A: Night splint + eccentric exercise	A: 5	12	A vs B	12wk: -1.09 (−2.41: 0.22)	
	B: Eccentric exercise	B: 6		VISA-A		
**HEEL BRACE**						
Knobloch 2008	A: Heel brace + eccentric exercise	A: 43	12	A vs B	12wk: -0.29 (−0.70: 0.12)	
	B: Eccentric exercise	B: 54		VASo		
Petersen 2007	A: Heel brace + eccentric exercise	A: 28	12	A vs B	6wk: ID	No difference between groups (p < 0.05)
	B: Eccentric exercise	B: 37		VASw	12wk: ID	
					54wk: ID	

CI = confidence interval; ID = insufficient data; NR = not reported; PES = Pain Experience Index; SD = standard deviation; SMD = standard mean difference; wk = week; VAS = visual analogue scale; VISA-A = Victorian Institute of Sports Assessment – Achilles; VISA-A-S Victorian Institute of Sports Assessment – Achilles Swedish; VASa = pain during activity; VASj = pain during jumping; VASm = pain in the morning; VASo = pain overall; VASs = pain after sport & recreation; VASw = pain during walking.

Evidence from meta-analysis of data from two studies comparing SWT to eccentric exercise [25,26] found no significant effects for outcomes of pain and function (VISA-A:–0.55,–2.21 to 1.11) at 16 weeks. One of these studies specified that they evaluated individuals with insertional AT [26], while the other studied individuals with midportion AT [25]. A further study by Rompe and colleagues [27] examined the effects of SWT when added to eccentric exercise, with effect sizes showing moderate significant effects favouring combined SWT and eccentric exercise over eccentric exercise alone after 16 weeks (–0.76,–1.28 to–0.24).

The 2007 study by Rompe et al. [25] also included a wait-and-see group, allowing comparisons to be made between SWT and a no-treatment control. Moderate significant effects were found that favour SWT at 16 weeks (−1.03,–1.62 to–0.44). Two studies [32,42] evaluated SWT using double-blind, placebo-controlled study designs, with effect sizes indicating similar outcomes. Costa et al. [32] compared SWT directly to application of sham SWT over 12 weeks. There were no significant pain effects favouring either SWT or sham at 12-week follow up (−0.44,–1.01 to 0.13). Although participants were followed up at 12 months, insufficient data was available to calculate effect sizes. Rasmussen et al. [42] investigated differences between SWT and sham SWT as an addition to a conservative therapy program that included eccentric exercise. After four weeks of intervention, no significant effects were found for either group (−0.52,–1.1 to 0.06). There was insufficient data to evaluate longer follow up periods.

Evidence from meta-analysis of data from two studies of higher methodological quality (10 and 12 out of 14) [43,45] does not support the use of laser therapy (LT) in conjunction with eccentric exercise. Both studies compared LT with sham LT, used in conjunction with eccentric exercise, and evaluated pain outcomes on a VAS over 12 weeks. Pooled data showed no significant effects at 4 weeks (−0.31,–1.88 to 1.26), but significant effects favouring LT were found at 12 weeks (−0.59,–1.11 to −0.07) (Figure 3).

Microcurrent therapy was investigated as an intervention for AT by one study [34] with a methodological quality rating of 8 out of 14. Chapman-Jones and Hill [34] compared a combined intervention of microcurrent therapy and eccentric exercise to eccentric exercise alone. While effect estimates were unable to be calculated, the authors reported significantly greater improvements in pain after 12, 26 and 52 weeks in favour of those receiving microcurrent therapy (*p* < 0.001).

*Braces and splints:* Meta-analysis was conducted using data from two studies that evaluated the addition of a night splint to an eccentric exercise program (PEDro scores 9 [47] and 6 [44] out of 14). Pooling of data for pain and function outcomes (VISA-A) showed no significant effects at 12 weeks (−0.35,–1.44 to 0.74).

**Figure 4:**
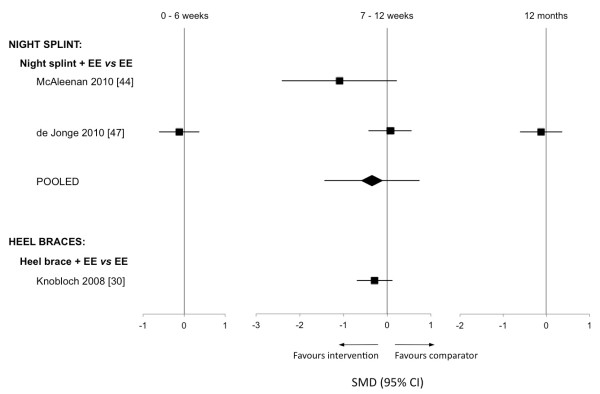
**Standardised mean differences for outcomes of pain with braces and splints.** EE = eccentric exercise

Two studies investigated the efficacy of a heel brace as an adjunct to eccentric exercise [30,31]. Both studies had methodological quality ratings of 6 out of 14 and, due to insufficient data provided by Petersen et al. [31], pooling of data was unable to be performed. Effect size calculations for Knobloch et al. [29] showed no significant effects for the addition of a heel brace to an eccentric exercise program at 12 weeks (−0.29,–0.70 to 0.12) (Figure 4). Petersen and colleagues [31] also reported no significant between-group differences over a one-year period.

## Discussion

Based on the available evidence, and limited opportunities for data pooling, it appears that a number of physical therapies may be effective in improving pain and function in those with AT. Effect sizes from multiple individual RCTs show eccentric exercise to be efficacious. Evidence from a meta-analysis indicates that SWT and eccentric exercise have similar effects. Pooled data also show LT to be more effective than sham when used in conjunction with eccentric exercise, however the addition of night splints to eccentric exercise does not provide any additional benefit. Microcurrent therapy may also be a worthwhile intervention for this condition, based on a single RCT. No evidence was found to support the use of heel braces as an adjunct to eccentric exercise.

One of the most interesting findings of this systematic review is that all but one of the included studies utilised an eccentric exercise program as their primary intervention, comparator intervention, or as a component of a multimodal approach. This, considered in conjunction with favourable findings regarding efficacy, suggests that eccentric exercise should be an integral component of AT management. The majority of studies that evaluated eccentric exercise as the primary intervention of interest utilised programs similar to the approach taken by Alfredson and colleagues [51]. This involves three sets of 15 repetitions of eccentric heel-drops twice daily for 12 weeks. An important component of Alfredson’s original program was that participants were encouraged to experience Achilles tendon pain during the exercise, an approach adopted by most studies (89%) [25-27,29-31,33,35,38-40,43-45,47]. This may influence participant compliance with the exercise program. However, participant adherence to eccentric exercise was monitored by few included studies, making it difficult to determine its role in the outcomes. The only studies to monitor compliance reported results ranging from 72 to 100% [45,47]. Methods such as information manuals, practical demonstrations or supervision were implemented by most studies to improve compliance. However, the consistent use of documentation, such as diaries, to measure compliance and determine its contribution to participant outcomes should be a feature of future studies.

Eccentric loading is currently recommended as the preferred exercise for tendinopathies, over other types of exercise such as concentric loading. However, differences in efficacy and therapeutic mechanisms between eccentric and other exercise are yet to be established. Rees and colleagues [52] compared eccentric and concentric loading of the Achilles tendon and found no differences in peak tendon force or length changes. However, high frequency oscillations were found to occur more commonly during eccentric than concentric loading. This difference has been proposed to achieve a greater therapeutic benefit by providing more stimulus for tendon remodelling [52]. This requires further investigation in order to establish a clearer understanding of the differences between eccentric and concentric tendon loading, and their relative efficacies for Achilles and other tendinopathies.

Outcomes of studies that compared eccentric exercise to no treatment or passive treatments provide important information regarding ideal management of AT. Findings of large significant effects favouring eccentric exercise over a wait-and-see approach [25] and cryotherapy [29] indicates that managing the condition in its chronic form by resting or using a cryotherapy program is inappropriate. This is consistent with beliefs that physical therapies such as eccentric exercise are needed to stimulate change within the tendon. Furthermore, the continuation of monitored tendon loading activities while undertaking an exercise rehabilitation program, which includes eccentric exercise, may provide no harm to patients. While findings of one study by Silbernagel and colleagues [39] supports this approach, further studies are needed to determine indications for continued tendon loading and its suitability for use with other interventions.

Overall findings regarding SWT need to be interpreted with caution. Although SWT appears more efficacious than no treatment [25], findings of two studies suggest that SWT is no more effective than a sham intervention [32,42], suggesting a placebo effect associated with pain and function outcomes. Furthermore, pooled study data reveals that SWT has a similar effect to eccentric exercise. It is only when SWT is used in conjunction with eccentric exercise that moderate effect sizes for pain and function are observed [27], suggesting that utilising SWT in combination with eccentric exercise is likely to produce superior patient outcomes than eccentric exercise or SWT alone. In comparison, LT was found to be an effective addition to eccentric exercise when compared to a sham intervention at 12 weeks, suggesting that it may be preferable to utilise LT rather than SWT as an adjunct to eccentric exercise. Interestingly, the two studies that compared LT to a sham intervention showed contrasting effect sizes at initial follow up (four weeks). This may be explained by methodological differences, where Tumilty and colleagues [43] utilised a shorter duration of treatment and application with lower power density and smaller spot size compared to Stergioulas et al. [43,45]. This reinforces the need for consensus regarding ideal LT application for AT, with particular consideration given to frequency, duration and dosage, and consistency when developing future LT protocols for clinical and research use.

There are important practical considerations when selecting SWT or LT as interventions for AT. Considering the need for access to specialised equipment as well as practitioner training, they may not represent an intervention with as widespread application and accessibility as eccentric exercise. However, it may be ideal for those who are unable or decline to use eccentric exercise. A further consideration is the discomfort that has previously been associated with SWT treatment [53]. Among the five studies using SWT, analgesia was not used in the preparation and the four (out of five) studies that monitored side effects did not report any significant adverse events, including pain. As such, although practitioners should always be aware of patient comfort during any treatment, it appears that SWT may not be pain provocative in all patients.

This systematic review identified no RCTs that have investigated night splints, heel braces, LT, or microcurrent therapy without eccentric exercise. It is therefore difficult to ascertain whether improvements in pain and function can be attributed to the intervention or the eccentric exercise program, which has established efficacy against a no-treatment control. As such, future studies are required to test these interventions in isolation in order to establish their efficacy as sole interventions for AT. Furthermore, other interventions that were not investigated by included studies, such as acupuncture, trigger point therapy, massage and foot orthoses may also be effective in the management of AT, and require investigation in RCTs.

While we did not restrict inclusion based on AT location, the majority of included studies that reported AT location utilised midportion tendinopathies only, followed by mixed midportion and insertional cohorts. Only one study investigated isolated insertional AT and the effects of SWT [26]. This is an important consideration for clinical application of these findings given that the location of symptoms may reflect different entities [54,55]. The insertion of the tendon has a tendency to develop cartilage-like or atrophic changes on the stress-shielded side of the enthesis as a result of reduced tensile load [56] and may explain why people with sedentary lifestyles develop insertional pathology. Thus, future studies should classify participants and report outcomes based on AT site to further increase knowledge regarding potential differences in treatment efficacy between midportion and insertional AT.

Age may also be important when selecting an appropriate intervention for AT. To our knowledge, the effect of age on outcomes of traditional interventions for AT has not been evaluated. The aging process results in collagen changes that may place humans at a higher risk of developing tendinopathies [57]. Weight bearing exercise has been shown to enhance the mechanical properties of tendons by increasing collagen synthesis [58]. However, the same process that may increase the risk of developing AT may also diminish the ability of the tendon to respond to exercise therapies. The studies that utilised participants with a mean age equal or less than 35 years all showed favourable effects for eccentric exercise when evaluating pain and/or function outcomes [29,46]. In comparison, only two of the five studies that utilised participants aged greater than 35 years (mean) favoured the eccentric exercise [25,38]. While it is important to consider that the control interventions were not consistent between studies, this does provide preliminary information to be considered for future studies.

The procedures adopted by this systematic review, including methodological quality ratings and data extraction, identified a number of features that should be addressed in future RCTs. Firstly, it is clear that more randomised studies that adhere to recommendations of the CONSORT statement [59] utilising appropriate control groups and blinding of participants and assessors whenever possible are required regarding physical therapies for AT. Secondly, consistent use of valid and reliable disease-specific outcome measures such as the VISA-A questionnaire [49] will facilitate comparisons between different studies, as well as further meta-analyses.

While this is the first systematic review on physical therapies for AT to utilise methodological quality ratings and conduct meta-analyses, there are limitations that must be acknowledged. Only English language studies were included, meaning that potentially relevant papers may have been excluded based on publication language. While three meta-analyses were performed, it was not possible to pool more than two studies per analysis. To achieve higher statistical power, it is necessary to pool a larger number of studies, which may be achieved by future systematic reviews on this topic [60]. Reviewers who rated studies on the modified PEDro scale were not blinded to author, institution and journal, and only one reviewer extracted study data, which may have introduced biases. Furthermore, the inclusion of studies with mixed locations of tendon pathology prevents this review from making clearer distinctions between the evidence for each entity, and, where possible, should be a consideration for future systematic reviews.

## Conclusions

This is the first systematic review of physical therapies for AT to perform meta-analyses and evaluate the methodological quality of included studies. Findings from individual RCTs support the use of eccentric exercise in the management of AT, with pooled data suggesting additional benefits using LT as an adjunct intervention, and similar outcomes when SWT is utilised as an alternative to eccentric exercise. There is emerging evidence supporting the use of microcurrent therapy in conjunction with eccentric exercises. It appears that continued tendon loading does not adversely affect pain and function outcomes. Sufficient evidence is lacking to enable recommendation of night splints and heel braces as a management option. Further high quality RCTs using disease specific outcome measures, consistent treatment protocols and reporting that adheres to the recommendations of the CONSORT statement are needed to clarify the clinical pathways for managing midportion and insertional AT.

## Abbreviations

AB, Andrea Bialocerkowski; AMI, Australian Medical Index; AOFAS, The American Orthopedic Foot and Ankle Society hindfoot scale; AT, Achilles tendinopathy; AUSPORT, Australian Sport Database; CI, Confidence interval; CINAHL, Cumulative Index to Nursing and Allied Health Literature; CONSORT, Consolidated Standards of Reporting Trials; EMBASE, Excerpta Medica Database; FILLA, Functional Index of the Leg and Lower Limb; KC, Kay Crossley; LT, Laser therapy; Medline, Medical Literature Analysis and Retrieval System; PEDro, Physiotherapy Evidence Database; PRISMA, Preferred Reporting of Systematic Reviews and Meta-analyses; RCTs, Randomised controlled trials; SD, Standard deviation; SSL, Samuel Sussmilch-Leitch; SMD, Standardised mean differences; SW, Stuart Warden; SWT, Shock wave therapy; VAS, Visual analogue scales; VISA-A, Victorian Institute of Sport Assessment-Achilles; κ, Kappa.

## Competing interests

Authors declare that they have no competing interests.

## Authors’ contributions

KC conceived of the review, participated in its design and coordination, screened titles, abstracts and full text articles for eligibility and drafted the manuscript. SSL participated in its design, performed the search strategy, appraised the methodological quality of studies, extracted study data and drafted the manuscript. AB participated in its design, appraised the methodological quality and contributed to the manuscript. SW screened titles, abstracts and full text articles for eligibility and contributed to the manuscript. NC participated in its design and drafted the manuscript. All authors read and approved the final manuscript.

## Supplementary Material

Additional file 1 Systematic review search strategy. Click here for file

Additional file 2 Modified PEDro scale for rating methodological quality. Click here for file
