# Supplementary material for: Physical therapies for Achilles tendinopathy: systematic review and meta-analysis
Source: J Foot Ankle Res. 2012 Jul 2;5:15. doi: 10.1186/1757-1146-5-15 (PMC3537637; doi:10.1186/1757-1146-5-15)
Supplement: Additional file 1 — Systematic review search strategy. [file 1757-1146-5-15-S1.pdf]

Additional file 1. Systematic review search strategy

	Combiners	Terms
1	Problem of interest	achill* OR triceps surae OR tendin* OR heel
2	Outcome	Pain
3		#1 AND #2
4	Intervention	ortho* OR electrotherapy OR therapeutic electric stimulation OR electric stimulation therapy OR heat OR cold OR ice OR inferential therapy OR laser OR exercise therapy OR rehabilitation OR tap* OR strapping OR eccentric* OR exercise therapy OR rehabilitation OR tap* OR strapping OR eccentric OR strengthening OR heel wedge OR heel raise OR heel brace OR shock wave therapy
5		#3 AND #4
	Limitations	English language, humans

Individual search terms were mapped to appropriate subject headings (MeSH)

* wild card/truncation (search term that begin with the letters preceding the asterisk)
