# Supplementary material for: Physical therapies for Achilles tendinopathy: systematic review and meta-analysis
Source: J Foot Ankle Res. 2012 Jul 2;5:15. doi: 10.1186/1757-1146-5-15 (PMC3537637; doi:10.1186/1757-1146-5-15)
Supplement: Additional file 2 — Modified PEDro scale for rating methodological quality. [file 1757-1146-5-15-S2.pdf]

Additional file 2. Modified PEDro scale for rating methodological quality

Criteria	Explanation
1. Eligibility criteria were specified	The source of subjects and a list of eligibility criteria must be included in the report.
2. Subjects were randomly allocated to interventions (in a cross over study, subjects were randomly allocated an order in which treatments were received)	The report must state that random allocation was used. The method for randomisation does not need to be described. Coin-tossing and dice-rolling are considered to be methods for random allocation. Allocation by hospital record number, birth date or alternation are considered quasi-randomisation procedures and do not satisfy this criterion.
3. Allocation was concealed	The person responsible for eligibility screening is unaware of subject's group allocation. The author is not required to directly state that allocation was concealed. The use of opaque envelopes or the requirement that subjects contact an off-site holder of the allocation schedule should be considered as concealed allocation.
4. The intervention groups were similar at baseline regarding the most important prognostic indicators	Reports must describe at least one measure of the severity of the condition and at least one key outcome measure at baseline. The same measure cannot be used for both severity and outcome. If differences in baseline measures for the groups differ, the rater must be satisfied that groups' outcomes will not differ by a clinically significant amount. The presentation of only baseline data is sufficient to satisfy this criterion.
5. There was blinding of all subjects	Refer to definition of blinding*
6. There was blinding of all therapists who administered the therapy	Refer to definition of blinding*
7. There was blinding of all assessors who measured one key outcome	Refer to definitions of blinding* and key outcome**
8. Measures of at least one key outcome were obtained from more than 85% of the subjects initially allocated to groups	Refer to definition of key outcome**
9. All subjects for whom outcome measures were available received the treatment or control condition as allocated or, where this was not the case, data for at least one key outcome was analysed by "intention to treat"	Refer to definition of key outcome**
10. The results of between-intervention group statistical comparisons are reported for at least one key outcome	Refer to definition of key outcome**
11. The study provides both point measures and measures if variability for at least one key outcome	Refer to definition of key outcome**
12. The sample is justified	Statistical evidence is provided to justify the sample size, power of the study and effect size.
13. The study uses outcome measures that have known validity and reliability	Evidence of reliability and validity of the study's outcome measures are provided. When more than one assessor is used for the outcome measures, an inter-rater reliability study should be performed and the results reported.
14. Adverse or side effects were reported	Adverse effects are reported to allocated treatment. If no adverse effects occur, this must be stated. A comparison of the intervention's beneficial effects and adverse effects should be performed and reported.
**Points allocation:** 1 point is awarded when a criterion is clearly satisfied. Points will not be awarded if it is possible that the criterion was not satisfied after conducting a thorough reading of the report.	

* Blinding occurs when the person (subject, therapist or assessor) is unaware of the group allocation. Subjects and therapists cannot be considered blind if they are able to distinguish between the treatments provided to each group. Where the key outcomes are self-reported, the assessor can be considered blind if the subject is blind.

** Key outcomes provide the primary measure of effectiveness of a treatment. More than one variable can be used as an outcome measure.
